# Full spectrum cannabidiol-rich extract reduced propofol dosage required for anesthetic induction in dogs—a pilot study

**DOI:** 10.3389/fvets.2024.1352314

**Published:** 2024-04-05

**Authors:** João Lourenço Hasckel Gewehr, Maria Laura Enzele, Lucas Marlon Freiria, Morgana Martins Nunes, Júlia Spengler, Ana Paula Dondoerfer Teixeira, Erik Amazonas, Vanessa Sasso Padilha

**Affiliations:** ^1^Veterinary Medicine, Federal University of Santa Catarina (UFSC), Curitibanos, Brazil; ^2^Veterinary Clinic School (CVE) of the Federal University of Santa Catarina (UFSC), Curitibanos, Brazil; ^3^Department of Biosciences and One Health (BSU), Center for Rural Sciences (CCR), Federal University of Santa Catarina (UFSC), Curitibanos, Brazil; ^4^Cannabis Development and Innovation Center (PODICAN/UFSC), Curitibanos, Brazil

**Keywords:** double blind, placebo controlled, prospective clinical study, cannabis, phytocannabinoid, anesthesia

## Abstract

**Introduction:**

Cannabinoids show great therapeutic potential, but their effect on anesthesia still remains unclear. Use of chronic recreational Cannabis in humans undergoing anesthetic procedures tends to require a higher dose when compared to non-users. On the other hand, studies on rodents and dogs have shown that cannabinoid agonists may potentiate certain anesthetics. This contrast of effects possibly occurs due to different time lengths of administration of different phytocannabinoids at different doses, and their distinct effects on the Endocannabinoid System, which is also affected by anesthetics such as propofol and isoflurane.

**Methods:**

Twenty-seven healthy male dogs, client-owned, ranging from 1 to 7 years, and from 5 to 35 kg were selected, mean weight 15.03±7.39 kg, with owners volunteering their animals to participate in the research performed in the Federal University of Santa Catarina (UFSC). Dogs were randomized into 3 groups. The Control Group (CON, *n* = 9), receiving only Extra Virgin Olive Oil, the same oil-base used in the treatment groups. Group 2 (G2, *n* = 9) received 2 mg/kg of total phytocannabinoids, and Group 3 (G3, *n* = 9) received 6 mg/kg of total phytocannabinoids. All groups received their treatments transmucosally, 75 min before their induction with propofol. Heart and respiratory rate, blood pressure, temperature and sedation were evaluated prior to, and at 30, 60, and 75  min after administration of the fsCBD-rich extract or Placebo extract. Preanesthetic medication protocol was also included across all treatment groups, 15  min before induction. Parametric data was analyzed with one-way ANOVA, followed by Student–Newman–Keuls (SNK) if significant statistical differences were found. Non-parametric data was analyzed using Friedman’s test, followed by Dunn test for comparisons between all timepoints in the same group. Kruskal-Wallis followed by Dunn was utilized for between groups comparisons. Propofol dose necessary for induction was analyzed through One-way ANOVA followed by Tukey’s Multiple Comparisons Test, using Instat by Graphpad, and differences were considered statistically significant when *p* < 0.05. Our analysis assessed if statistical significance was present between time points in the same group, and between groups in the same time points.

**Results:**

In our study, 6  mg/kg of total phytocannabinoids were able to reduce the dose of propofol necessary for induction by 23% when compared to the control group. The fsCBD-rich extract did not produce significant sedation within or between groups, although statistically significant differences in heart rate and systolic blood pressure were found.

**Discussion:**

Our findings indicate that phytocannabinoids could be an adjunct option in anesthesia, although further research is necessary to better confirm this data. Additionally, further research is needed to determine the best dosage, delivery method, time for administration, ideal molecular profile for desired effects, safety, drug–drug interactions, and transurgical effects.

## Introduction

Cannabis criminalization has made it challenging for scientists to study cannabis, limiting the scientific and medical communities’ understanding of its therapeutic potential. Modern principles of anesthesiology were established during cannabis prohibition and, for great part, under a complete unawareness of the endocannabinoid system and the role it may play in anesthesia. Therefore, the Endocannabinoid System (ECS) relation with anesthetics has not yet been deeply studied and remains unclear. There is, however, growing evidence that many commonly utilized pharmaceuticals interact with the ECS (1–7) and therefore many of their mechanisms may be, at least partially, mediated through this system.

Most modern research focused on the impact of chronic marijuana use on anesthetic induction In humans with chronic use of high doses of Tetrahydrocannabinol (THC), an exogenous agonist of the cannabinoid receptor 1 (CB1), a higher dose of propofol seems to be needed when compared to non-users (8–10), possibly due to a desensitization or downregulation of the receptor, which has been reported in rodents and humans chronically exposed to high levels of this molecule. In contrast, acute administrations of THC appear to increase the density of these receptors, and improve their sensitivity to endocannabinoids (4). Research done in mice has demonstrated that the administration of a synthetic cannabinoid agonist actually reduced propofol doses (1). This was also reported in dogs receiving phytocannabinoids intraperitoneally (11).

These different effects may be caused by different time lengths of administration of THC, although this awaits further research. Most likely, an increase in the receptor density and/or sensitivity improve propofol effects, since studies have indicated that propofol indirectly modulates CB1 (1–3, 7).

The CB1 receptor is classified as a G-protein coupled receptor (GPCR) that interacts with ion channels in presynaptic neurons leading to its control on neurotransmission and pain modulation (12, 13). One of the endogenous ligands of the CB1 receptor is the N-arachidonoylethanolamine or anandamide (AEA), transported intracellularly by Fatty Acid Binding Proteins (FABP) and later catabolized by the Fatty Acid Amide Hydrolase (FAAH) enzyme. Propofol appears to be a FAAH inhibitor (1, 3, 14), so by reducing the reuptake of anandamide, propofol allows a higher availability of this molecule. Cannabidiol (CBD), a phytocannabinoid, also inhibits this enzyme and binds to FABPs, reducing AEA breakdown and reuptake. Many of the effects of CBD are likely also mediated through these mechanisms of action (15). Besides enzymatic inhibition, CBD also acts as an allosteric modulator at this receptor, which can also be one of its mechanisms (16).

In this research, we evaluated the effects of different doses of phytocannabinoids administered transmucosally 60 min before anesthetic premedication in the propofol doses necessary for anesthetic induction.

## Materials and methods

### Full spectrum cannabidiol-rich extract and ethics board approval

The Cannabis extracts (fsCBD-rich extract) used in this study were provided by the “Cannabis Sem Fronteiras” through their veterinary phytotherapy extension project (number 202213421). The oil consisted in a full spectrum cannabidiol-rich extract (fsCBD-rich) of CBD:THC ratio of approximately 21:1, and a CBD:CBDa ratio of 1.6:1 (Certificate of Analysis THCA22021606-01, The Higher Commitment Analytical Lab, United States) (Table 1). The research was approved by the University Ethics Committee (number 2065211122), and the authorization to possess, transport and research Cannabis extracts was granted by means of the *habeas corpus* n° 5040089-27.2021.4.04.7200/SC.

**Table 1:** fsCBD-rich extract composition.

Analyte	Result (mg/g)	Result (%)
THCa	1.10	0.11
Δ9-THC	36.27	3.627
Δ8-THC	ND	ND
THCv	ND	ND
CBDa	304.72	30.472
CBD	517.73	51.772
CBN	0.79	0.079
CBGa	3.60	0.36
CBG	0.69	0.069
CBC	23.65	2.365
Total CBD	784.970	78.50%
Total THC	37.237	3.72%
Total cannabinoids	850.497	85.05%

### Animals

Twenty-seven healthy male dogs, client-owned, ranging from 1 to 7 years, and from 5 to 35 kg were selected, mean weight 15.03 ± 7.39 kg, with owners volunteering their animals to participate in the research performed in the Federal University of Santa Catarina (UFSC). Dogs were randomized into 3 groups. The Control Group (CON, *n* = 9), receiving only Extra Virgin Olive Oil, the same oil-base used in the treatment groups. Group 2 (G2, *n* = 9) received 2 mg/kg of total phytocannabinoids, and Group 3 (G3, *n* = 9) received 6 mg/kg of total phytocannabinoids. All groups received their treatments transmucosally, 75 min before their induction with propofol.

### Inclusion and exclusion criteria

Upon arrival at the Veterinary Clinic School located at the Federal University of Santa Catarina - Curitibanos Campus, animals were physically examined and assessed for their level of activity and consciousness, mucosal color, capillary filling time, lymph node size, hydration, heart and respiratory rate, and rectal temperature by a veterinarian. Blood was drawn from each patient and sent to the Laboratory of Clinical Analysis of UFSC (LACLIN). Hematological and biochemical evaluation including complete blood count, leukogram, Alanine Aminotransferase (ALT), Alkaline Phosphatase (ALP), urea, creatinine, albumin and total plasma protein levels. Animals that had slight elevations in albumin and total plasma protein which appeared clinically insignificant were not excluded. Animals that had any evidence of heart, liver, kidneys or metabolic disease in the blood work or physical examination were excluded from the study.

### Randomization and blinding

Animals were randomly assigned to one of the three groups by the research coordinator through the randomizer.org website. Only the coordinator and the trained personnel administering the oil were aware of which treatment group each patient belonged to.

### Clinical trial

Animals that met the criteria for inclusion were selected to participate in the research. Owners were requested not to provide food or water to the animals 12 h before the procedure. Upon arrival, hair was removed from the venous access region and in the region used to assess systolic blood pressure, and the patients were allowed to be accustomed to the dog kennels of University School Clinic (CVE/UFSC) for 10 min before their first evaluation.

Patients were evaluated in seven time points: immediately before the administration of the CBD-rich extracts (T1), 30 min after fsCBD-rich extract administration (T2), 60 min after fsCBD-rich extract administration (T3), 15 min after the administration of the preanesthetic medication (PM) (T4), at their venous access (T5), at their anesthetic induction (T6) and at their intubation (T7).

The fsCBD-rich extract and placebo were applied to patients’ gums by the same trained personnel immediately after the evaluations at T1, and in between evaluations, the animals were left alone in a quiet, slightly dark kennel.

Evaluations performed at T1, T2, T3, and T4 consisted of sedation level via the validated abbreviated Wagner scale (17), consisting in 4 evaluations: Spontaneous posture (E1), eyeball position (E2), noise reaction (E3), and attitude (E4). During all of these time points, vital parameters (VP) including heart rate (HR) and respiratory rate (RR), systolic blood pressure (SBP), and rectal temperature (RT) were also assessed by the same veterinarian. HR was assessed through auscultation with a stethoscope, RR was assessed through visual inspection, SPB was evaluated through a Parks Model 811-B Doppler, using cuffs of a size consistent with the animal being evaluated (40–50% of the circumference of the animal’s limb), and RT using a thermometer.

Immediately after the VP assessment at T3, PM was administered. It consisted of 0.05 mg/kg of acepromazine and 0.2 mg/kg of methadone administered intramuscularly, 60 min after the administration of the fsCBD-rich extract. After the evaluations at T4, animals were brought to the surgery center, in the next room, ready for their anesthetic induction. Venous access was obtained by placement and fixing of an intravenous catheter in the left thoracic limb, and its resistance evaluated through the scale adapted from Bortolami et al. (18), consisting of evaluation 5 at T5.

Immediately after, propofol was administered intravenously at a rate of 2 mg/kg/min through a Digicare syringe infusion pump, model SR31x. Administration stopped when the patient lost palpebral reflex and mandibular tone, allowing intubation, consisting in the Evaluation 6 (E6) at T6. Intubation was performed using an endotracheal tube of a compatible size, and its ease of placement was assessed using the modified scale of Lerche et al. (19), being the evaluation 7 (E7), performed at T7. Patient assessments scales are shown in Table 2. Timepoints are summarized in Table 3.

**Table 2:** Patient assessments scales.

A. Sedation score [abbreviated scale of Wagner et al. (17)]	
**E1—Spontaneous posture**	
Standing (all limbs supported)	0
Tired, but standing	1
Laying, but able to stand up	2
Laying, but has difficulty standing up	3
Unable to stand up	4
**E2—Eye position**	
Centralized	0
Rotated rostrally, but not covered by the Nictitating membrane	1
Rotated rostrally and covered by the Nictitating membrane	2
**E3—Noise reaction (hand claps)**	
Normal reaction (turns head toward the noise/moves away)	0
Reduced reaction (reduced turning of the head, reduced avoidant movement)	1
Minimal reaction	2
No reaction	3
**E4—Attitude**	
Excited	0
Alert/normal	1
Quiet	2
Stupor	3
**B. Bortolami et al.** (18) **scale (adapted)**	
**E5—Resistance to catheter insertion**	
Animal in sternal position, no reaction	0
Mild movement of the limb and muscular tension, with minimal restraint necessary	1
Limb pull away, attempt to get away, moderate restraint necessary	2
Attempts to escape, vocalization, aggressive behavior, significant restraint necessary, multiple placement attempts	3
**C. Lerche et al.** (19) **scale (adapted)**	
**E7—Ease of orotracheal intubation after anesthetic induction**	
Intubation successful in one attempt, without signs of laryngospasm or physical movement	0
Intubation successful in one attempt, with signs of physical movement, such as cough	1
Intubation successful in more than one attempt, with or without physical movement	2
Intubation unsuccessful due to excessive mandibular tone, laryngospasm, or physical movement	3

A. Sedation score [abbreviated scale of Wagner et al. (17)]; B. Resistance to catheter insertion [Adapted from Bortolami et al. (18)]; (C). Ease of orotracheal intubation [Adapted from Lerche et al. (19)].

**Table 3:** Timepoints evaluation summary.

	Timepoints of patient assessment						
	T1	T2	T3	T4	T5	T6	T7
Timing	Before CBD	30′ after CBD	60′ after CBD	75′ after CBD 15′ after PM	Immediately after T4	Immediately after T5	Immediately after T6
Interventions	Wagner scale evaluation (E1, E2, E3, E4) VP assessment CBD administration	Wagner scale evaluation (E1, E2, E3, E4) VP assessment	Wagner scale evaluation (E1, E2, E3, E4) VP assessment PM administered	Wagner scale evaluation (E1, E2, E3, E4) VP assessment Animal brought to the surgery center	Venous access was obtained Bortolami et al. evaluation (E5)	Anesthetic Induction Propofol volume assessed (E6)	Patient Intubated Ease of intubation assessed through adapted scale of Lerche et al. (E7)

Immediately after induction and intubation, the animals were coupled to the anesthetic system consistent with their size. Anesthetic maintenance was carried out through the administration of the volatile anesthetic Isoflurane dose-effect by a universal vaporizer, diluted in 100% O^2^. Patient vital signs were evaluated through pulse oximeter and rectal thermometer linked to a Deltalife multiparametric monitor, the respiratory rate was monitored by visualizing the patient’s chest movement, and his systolic blood pressure was measured using a Parks Model 811-B Doppler with a compatible cuff size, every 5 min or when necessary.

Patients were considered in the appropriate surgical anesthetic plane when they displayed no palpebral reflex, no mandibular tone and eye globes rotated rostrally. Hair was removed from the surgical site, and then prepared with aseptic technique with alcohol- iodine-alcohol, and local block performed with 2% lidocaine, at a dose of 5 mg/kg, applied to the spermatic cord. Subsequently, the open orchiectomy technique was performed by three surgeons throughout the project. At the end of the procedure, the isoflurane was turned off, and the patient was extubated when a swallowing reflex was present.

After the completion of the surgical procedure, patients were medicated subcutaneously with 0.2 mg/kg of Meloxicam, 5 mg/kg of Enrofloxacin and 25 mg/kg of dipyrone. After patient’s regained consciousness with a stable body temperature and normal coordination, medical release to the client was performed, with instructions for the use of an Elizabethan collar until the non-absorbable skin stitches were removed. The patients were prescribed Meloxicam 0.1 mg/kg for and Dipyrone 25 mg/kg, both orally, for 3 and 5 days, respectively.

Most patients returned after 10–15 days for suture removal or follow-up. A few owners decided to remove the sutures in private clinics for convenience, but reported back to the researchers indicating the animals were well, with no apparent complications from the surgical procedure. A few patients had suture dehiscence due to owners not being consistent with the use of the Elizabethan collar. Owners did not report any other major post-surgical complications.

### Statistical analysis

Parametric data was analyzed with one-way ANOVA, followed by Student–Newman–Keuls (SNK) if significant statistical differences were found.

Non-parametric data was analyzed using Friedman’s test, followed by Dunn test for comparisons between all timepoints in the same group. Kruskal-Wallis followed by Dunn was utilized for between groups comparisons. Differences were considered statistically significant when *p* < 0.05, and all these analyses were done using Instat by Graphpad.

Propofol dose necessary for induction was analyzed through One-way ANOVA followed by Tukey’s Multiple Comparisons Test, using Instat by Graphpad.

Our analysis assessed if statistical significance was present between time points in the same group, and between groups in the same time points. This way, we assessed if groups receiving fsCBD-rich extract showed any signs of sedation after administration, as well as if they appeared more sedated after the administration of the preanesthetic medication when compared to the control group.

Intraclass correlation coefficient was done comparing the results of both evaluators, assessing the reliability of the scale.

## Results

### Sedation scale

**Figure 1:**
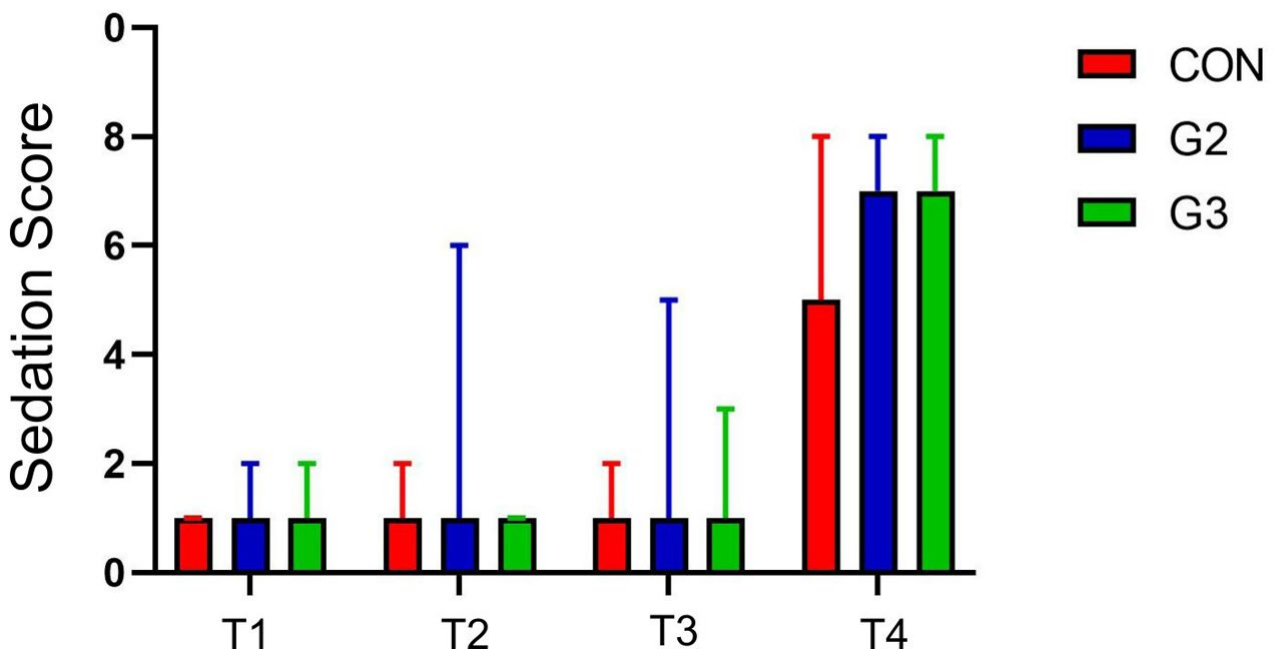
Sedation score (sum of E1, E2, E3, and E4 of the abbreviated Wagner Scale) by timepoint for all groups: treated with control (CON), 2 mg/kg (G2) and 6 mg/kg (G3) of total phytocannabinoids, respectively, 75 min before anesthetic induction with propofol.

The sedation chart for all groups can be found in Figure 1. Although both evaluators were veterinarians, evaluator 2 is a postgraduate in anesthesiology, and is considered more experienced than Evaluator 1.

The G3 group, receiving 6 mg/kg of phytocannabinoids, required a significantly lower propofol dose (*p* < 0.0138) when compared to the placebo group (CON), by way of a one-way ANOVA followed by Tukey’s multiple comparisons test.

Statistical significance was found within all groups (*p* value < 0.0001), when comparing E1, E2, E3 and E4 at T1, T2, T3 with T4 through one-way ANOVA and Student–Newman–Keuls (SNK) Multiple Comparisons Test. Comparisons involving only T1, T2, and T3, however, have not shown significant statistical difference, in all groups. Considering T4 was 15 min after the administration of the preanesthetic medication, sedation was expected for all patients.

Comparisons of the scores of E1, E2, E3, and E4 on T1, T2, and T3 among different groups have not shown significant statistical differences using one-way ANOVA analysis, for both evaluators. T2 had statistical difference utilizing this test, but SNK Multiple Comparisons Test demonstrated *p* value as 0.0936, considered not significant. Analysis of the total scores (sum of all evaluations performed on that timepoint) between groups have not demonstrated statistical significance.

These results indicate that administrations of 2 mg/Kg or 6 mg/Kg of phytocannabinoids in this fsCBD-rich extract did not have significant sedative effects on the patients, nor did they amplify the sedative effects of the combination of Acepromazine and Methadone.

### Respiratory frequency

**Figure 2:**
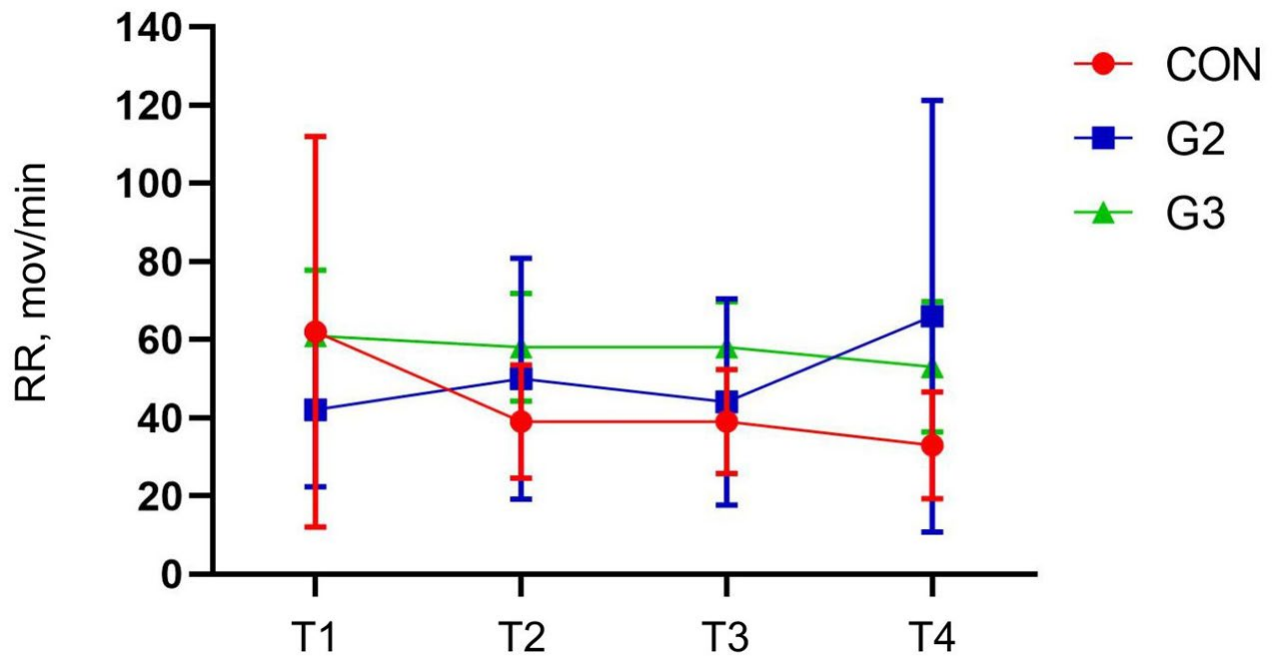
Mean values and standard deviation of the respiratory rate (RR, mov/min) of dogs underwenting elective orchiectomy, and treated with control (CON), 2 mg/kg (G2) and 6 mg/kg (G3) of total phytocannabinoids, respectively, 75 min before anesthetic induction with propofol.

One-way ANOVA and SNK Multiple Comparisons tests have not demonstrated statistical difference in the respiratory frequency between evaluation time points, in all groups (Figure 2).

### Heart rate

**Figure 3:**
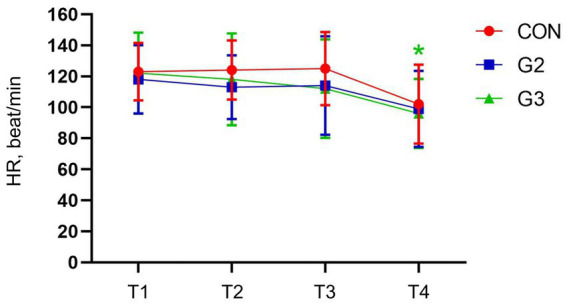
Mean values and standard deviation of the heart rate (HR, beat/min) of dogs underwenting elective orchiectomy, and treated with control (CON), 2 mg/kg (G2) and 6 mg/kg (G3) of total phytocannabinoids, respectively, 75 min before anesthetic induction with propofol.

Analysis through one-way ANOVA had statistical differences in CON and G3. SNK Multiple Comparisons test, however, has not shown statistical difference for CON but has confirmed the statistical significance in the heart rate between T1, T2, and T3 with T4 in the G3 (Figure 3).

### Systolic blood pressure

**Figure 4:**
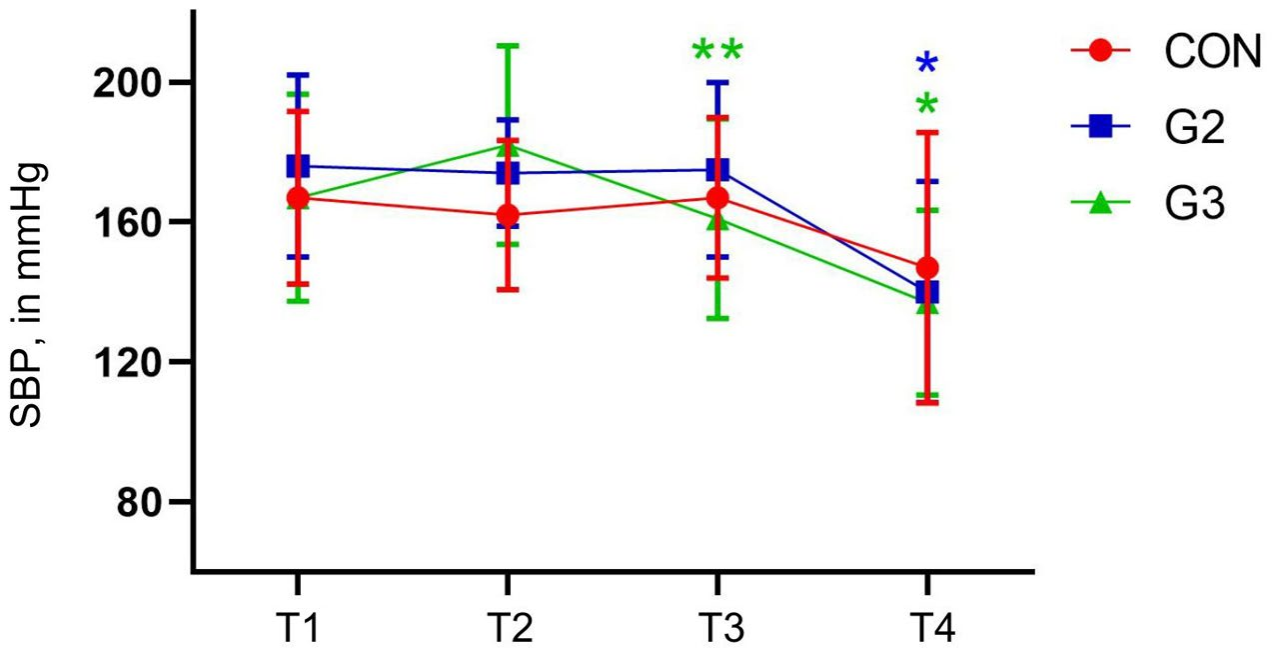
Mean values and standard deviation of the systolic blood pressure (SBP, in mmHg) of dogs underwenting elective orchiectomy, and treated with control (CON), 2 mg/kg (G2) and 6 mg/kg (G3) of total phytocannabinoids, respectively, 75 min before anesthetic induction with propofol.

No statistical differences between evaluation time points in the CON. G2, however, had significant differences between T1, T2, T3 with T4. G3 had the same differences as G2, and also between T1 and T2, before the administration of the preanesthetic medication. All these statistical significant differences were confirmed through SNK Multiple Comparisons test (Figure 4).

### Temperature

**Figure 5:**
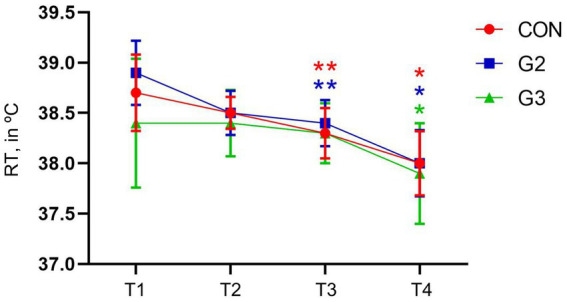
Mean values and standard deviation of the Rectal Temperature (RT, in °C) of dogs underwenting elective orchiectomy, and treated with control (CON), 2 mg/kg (G2) and 6 mg/kg (G3) of total phytocannabinoids, respectively, 75 min before anesthetic induction with propofol.

Statistical differences were found in all groups, between T1, T2, T3 with T4. CON also had a significant difference between T1 and T3, and G2 had a significant difference between T1 and T2, and T1 and T3, confirmed through SNK Multiple Comparisons Test (Figure 5).

Mean values and standard deviation for all VP evaluated can be found in Table 4.

**Table 4:** Mean values and standard deviation of heart rate (HR, beat/min), respiratory rate (RR, mov/min), systolic blood pressure (SBP, in mmHg) and rectal temperature (TR in °C) of dogs underwent elective orchiectomy, and treated with placebo (CON), 2 mg/kg (G2) and 6 mg/kg (G3) of total phytocannabinoids, respectively, 75 min before anesthetic induction with propofol.

		T1	T2	T3	T4
HR (beat/min)	CON	123 ± 18.59	124 ± 19.07	125 ± 23.58	102 ± 25.53
	G2	118 ± 22.01	113 ± 20.50	114 ± 31.74	99 ± 24.47
	G3	122^a^ ± 26.21	118^a^ ± 29.73	112^a^ ± 31.83	96^b^ ± 22.34
RR (mov/min)	CON	62 ± 49.96	39 ± 14.49	39 ± 13.33	33 ± 13.71
	G2	42 ± 19.79	50 ± 30.86	44 ± 26.44	66 ± 55.24
	G3	61 ± 16.85	58 ± 13.85	58 ± 11.68	53 ± 16.73
SBP (in mmHg)	CON	167^a^ ± 24.69	162a ± 21.36	167^a^ ± 22.91	147^a^ ± 38.69
	G2	176^ab^ ± 26.02	174^ab^ ± 15.24	175^ab^ ± 24.88	140^b^ ± 31.61
	G3	167^ab^ ± 29.55	182^ab^ ± 28.53	161^ab^ ± 28.59	137^ab^ ± 26.41
RT (in °C)	CON	38.7 ± 0.38	38.5 ± 0.16	38.3 ± 0.25	38 ± 0.32
	G2	38.9 ± 0.32	38.5 ± 0.22	38.4 ± 0.23	38 ± 0.33
	G3	38.4 ± 0.64	38.4 ± 0.33	38.3 ± 0.30	37.9 ± 0.50

### Resistance to catheter insertion

No statistical differences were found in analysis between groups through One-Way ANOVA.

### Anesthetic induction

**Figure 6:**
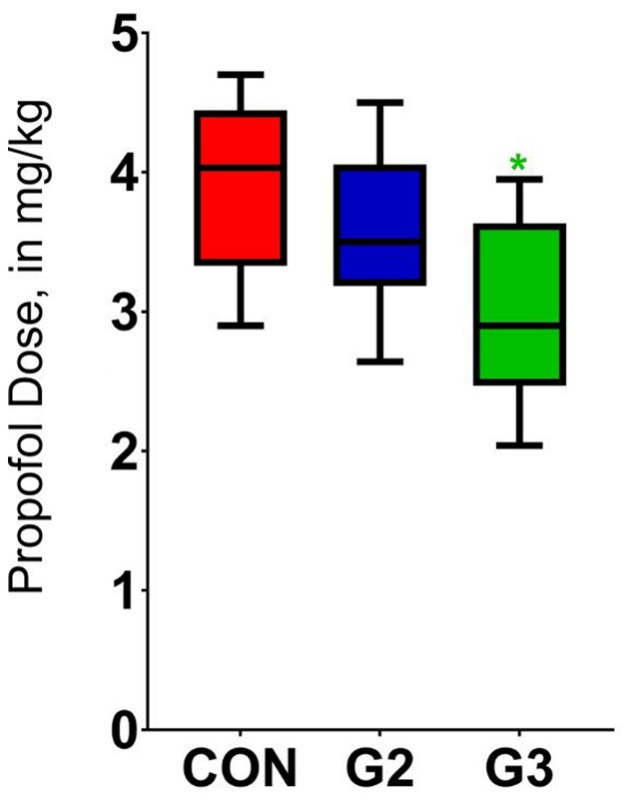
Mean values and standard deviation of the propofol dose required for anesthetic induction of dogs underwenting elective orchiectomy, and treated with control (CON), 2 mg/kg (G2) and 6 mg/kg (G3) of total phytocannabinoids, respectively, 75 min before anesthetic induction with propofol.

Analysis of the Propofol dosage required for induction showed a significant statistical difference among G3 and CON (*p* < 0.0138) using Tukey multiple comparison test. Analysis between G3 and G2, and between G2 and CON have not demonstrated significant statistical differences (Figure 6).

### Ease of intubation

No statistical differences were found in analysis between groups through One-Way ANOVA.

Means of the patients weights, propofol dose and volume, as well as medians of the scores of their resistance to catheter insertion and ease of intubation can be found in Table 5.

**Table 5:** Means of the patients weights, propofol dose and volume, and medians of the scores of their resistance to catheter insertion and ease of intubation.

Groups	Weight (kg) Mean ± SD	Catheter resistance (Median ± SD)	Ease of intubation Median ± SD	Propofol (mL) Mean ± SD	Propofol (mg/kg) Mean ± SD
Control	14.06 ± 7.28	0.4 ± 0.72	0.1 ± 0.3	5.34 ± 2.44	3.88a ± 0.62
G2 (2 mg/kg)	15.31 ± 9.61	0.4 ± 1.01	0.5 ± 0.72	5.12 ± 2.69	3.6ab ± 0.57
G3 (6 mg/kg)	15.83 ± 5.5	0.1 ± 0.33	0.1 ± 0.33	5.01 ± 1.61	2.99b ± 0.63

### Transsurgical parameters

During the surgery, patients were assessed using a pulse oximeter and rectal thermometer linked to a Deltalife multiparametric monitor (DL1000). Since anesthetic maintenance was done with Isoflurane dose-effect administered with an universal vaporizer under the supervision of an anesthesiologist, there was no standardization and measurement of the concentration utilized. Empirically, there was no evidence of any trans surgical problematic issues for any group.

## Discussion

The Cannabis plant has been utilized across various human cultures since ancient times, serving numerous purposes including medicine, fiber production, food, recreational use, and religious ceremonies (20). Veterinary literature reports the historical use of cannabis in animals dating back to at least the twelfth century (21), with early investigations focusing particularly on its effects in dogs (22). Despite extensive historical usage, gaps remain in our understanding of cannabis-derived molecules, particularly concerning their impact during anesthesia. Contemporary research has predominantly examined the anesthetic implications of marijuana consumption in humans, revealing altered propofol requirements in chronic users (8–10). Phytocannabinoids like THC and CBD exert a range of effects through diverse mechanisms (4, 15), offering therapeutic potential across multiple medical domains. Furthermore, both propofol and CBD inhibit the enzyme FAAH (1–3, 7, 14, 15), leading to increased levels of the endocannabinoid Anandamide, suggesting a shared pharmacological mechanism that may potentiate the effects of propofol through the endocannabinoid system.

In this study, a single-dose of 6 mg/kg of the fsCBD-rich extract produced a reduction of 23% on propofol dose necessary for induction in dogs. This finding agrees with other research that has shown that a *Cannabis indica* extract administered intraperitoneally in dogs reduced the propofol dosage for induction (11). Although authors have mentioned ropy saliva, this was not observed in the current study. Findings in rodents further confirm these results in which synthetic cannabinoid agonists reduced propofol dosage, while antagonists had the opposite effect (1).

Sedation scores comparison did not have significant statistical differences between groups, and between T1, T2 and T3, for both evaluators. These findings suggest that the administration of 2 mg/Kg or 6 mg/Kg of total phytocannabinoids from the fsCannabidiol-rich extract used in this study did not induce significant sedative effects on the patients, nor did they enhance the sedative effects of the combination of acepromazine and methadone.

CBD is not described as exercising sedative or psychoactive effects, with studies reporting doses ranging from 8 mg/kg (23) and up to 100 mg/kg (24) in dogs without major side effects. THC has been described as causing neurological side effects, such as hyperesthesia and proprioceptive deficit (25), as well as ataxia, lethargy, and hypothermia (26). These effects are described as temporary, more likely to appear when THC is in higher amounts and in first experience as patients seem to create a tolerance to these side effects when the dosage is incremented gradually over time. This molecule is a partial agonist of CB1 receptors located in the nervous system, and its short-term administration increases their expression, as well as improving the binding affinity of endocannabinoids therein (4).

THC was the third most concentrated molecule in the fsCBD-rich extract in a CBD:THC ratio of approximately 21:1. Although rare, side effects have been described for doses of THC of 0.1 mg/kg (26). In our study, G2 received 0.09 mg/kg and G3 received 0.28 mg/kg of THC, and our team has not seen any neurological side effects described by previous authors. The second most concentrated molecule in the extract used in this research was Cannabidiolic Acid (CBDa), present in a CBD:CBDa ratio of 1.6:1. Research in rodents demonstrates that CBDA appears to enhance CBD bioavailability by up to 14 times (27), and that its presence in the extract also improved bioavailability in dogs (28, 29).

The presence of CBDa and THC may be responsible, at least partially, of the results we have described in our study. Both molecules might have altered the pharmacodynamic and/or pharmacokinetic of CBD, and THC might have increased CB1 receptor expression, potentiating propofol indirect action on it. Nevertheless, these are the authors hypothesis, and still await further research.

In the present study, the fsCBD-rich extract was administered to the patients’ gums. Oral and transmucosal administration have practically identical pharmacokinetics (PK), indicating that it is absorbed through the gastrointestinal tract in both routes of administration (28). Although there are discrepancies between the pharmacokinetics of CBD depending on its formulation and physiological factors, research indicates that its half-life is 4–8 h, and its plasma peak is reached within 2 h (23, 28). A review published in 2023 has stated that Cannabis products have a large variability, and these results should be interpreted carefully (29). Nevertheless, the administration time of the extracts was calculated based on these PK studies, in which the administration of the PM and propofol coincided with the plasma peak of phytocannabinoids.

Sedation scores analysis have demonstrated statistical significance (*p* value > 0.0001) when comparing T1, T2, and T3 with T4, for both evaluators. That was expected given that T4 occurred 15 min after administering preanesthetic medication, thus sedation was anticipated for all animals, considering that acepromazine and methadone can produce effective sedation when associated in adequate doses (30).

The Abbreviated Wagner Sedation scale was consistent in describing sedation for both evaluators after PM, as designed to. Despite our results not showing an enhanced sedation for the association of phytocannabinoids and PM, this could be a limitation of the Sedation scale that was utilized. Its parameters might not have been representative to evaluate the changes in animal’s behavior that the fsCBD-rich extracts produced, since anxiolytic effects have already been described (31, 32).

G2 and G3 showed a statistically significant reduction in HR and SBP between T0, T1 and T2 with T3, and G3 also showed a reduction in SBP between T1 and T2, which did not occur in the CON. These variations in these parameters may have occurred due to the administration of the fsCBD-rich extract, suggesting possible anxiolytic effects. To better evaluate these results, it is likely that a specific scale for this type of analysis would be more appropriate.

These findings are corroborated by the literature, which indicates that Cannabis extracts can cause a decrease in HR and SBP in animals in stressful situations (31–34).

Other studies mention a modest change in heart rate variability and decreased heart rate after acute ingestion of CBD in humans, pointing out the inconsistency of this information across different studies. This data inconsistency can occur both due to individual factors and variations in product presentations (35). These changes in HR and SBP may be transient and occur quickly, and may not be observed in long-term studies depending on the time of assessment (33).

No statistically significant differences were observed in RR when comparing timepoints and groups. A systematic review mentions discrepant information on the hemodynamic effects of CBD, where it reduces RR in times of stress, but not under controlled conditions, in several species (34).

All groups showed a significant reduction in RT. Hypothermia is described as one of the side effects of delta-9-THC, especially at higher doses, while CBD has not been shown to cause changes in body temperature (26). The ECS is related to the maintenance of body temperature (36).

On the other hand, the area where patients were kept between assessments did not have thermal control, being necessary to use a portable heater to keep the environment warm as this research was done during the winter. This lack of precise temperature control may have been a significant factor in the reduction in RT identified in all groups participating in the research.

The analysis of HR, RR, SBP, and RT between groups, on the other hand, did not demonstrate a significant difference.

In conclusion, we have found that fs-CBD-rich Cannabis extracts have the potential of being an adjunct in anesthetic protocols, although this is a very initial finding. We strongly encourage other researchers to further investigate these results and its mechanisms, as understanding the interactions between the anesthetics, the Endocannabinoid System, and their interaction with phytocannabinoids, may allow discoveries in underlying mechanisms of different pharmaceuticals, and the development of new drugs or protocols that improves anesthetic procedures.

### Limitations

Animals had different temperaments, with standardization not being ideal. Although this effect is minimized by the randomization and all of them were docile, this personality difference was evident throughout the research, as some animals were not accustomed to medical personal handling as the others. Animals also did not have a long period of time to be accustomed to the dog kennels, and only male dogs were included in this study.

The room which patients were kept between evaluations did not have controlled temperature. A portable heater had to be used in order to keep the patients warm during winter temperatures, and these different temperatures could be a factor in their behavior, and in the changes observed in heart rate, systolic blood pressure and temperature.

The Federal University of Santa Catarina lacks equipment to analyze how much volatile anesthetics are being used in each patient, with dose-effect being the standard procedure. Without knowing precisely how much volatile anesthetic was being used during the surgery, our research was unable to make any conclusions on the effects of the combination of Isoflurane and the fsCannabidiol-rich extract. The trans surgical parameters need to be better examined in future, better controlled studies.

Capnography parameters were not assessed among groups, as the Federal University of Santa Catarina does not have a calibrated vaporizer, capnography and gas analyzer available. With this being one of the main limitations of this research, and although all patients were well at the end of the procedures, we strongly advocate for other researchers to confirm all of the data here described, and further investigate the safety of the administration of full scale cannabidiol-rich extracts before general anesthesia through more robust, well controlled studies.

## Data availability statement

The original contributions presented in the study are included in the article/supplementary material, further inquiries can be directed to the corresponding author.

## Ethics statement

The animal studies were approved by the Animal Ethics Board of the Federal University of Santa Catarina (number 2065211122). The studies were conducted in accordance with the local legislation, institutional requirements and international ethical research guidelines. Written informed consent was obtained from the owners for the participation of their animals in this study.

## Author contributions

JH: Conceptualization, Formal analysis, Funding acquisition, Investigation, Methodology, Project administration, Resources, Supervision, Writing – original draft, Writing – review & editing. ME: Data curation, Project administration, Supervision, Writing – review & editing. LF: Project administration, Writing – review & editing. MN: Writing – original draft. JS: Writing – original draft. AD: Writing – original draft. EA: Conceptualization, Data curation, Investigation, Methodology, Project administration, Resources, Supervision, Writing – original draft, Writing – review & editing. VS: Conceptualization, Data curation, Formal analysis, Investigation, Methodology, Project administration, Supervision, Validation, Writing – original draft, Writing – review & editing.
